# Parametric Simulations of Slanted 1D Photonic Crystal Sensors

**DOI:** 10.1186/s11671-016-1321-0

**Published:** 2016-03-22

**Authors:** Aaron Breuer-Weil, Naif Nasser Almasoud, Badaruddin Abbasi, Ali K. Yetisen, Seok-Hyun Yun, Haider Butt

**Affiliations:** Nanotechnology Laboratory, School of Engineering, University of Birmingham, Edgbaston, Birmingham, B15 2TT UK; College of Dentistry, University of Dammam, Dammam, Saudi Arabia; Deanship for Scientific Research, University of Dammam, Dammam, Saudi Arabia; Harvard Medical School and Wellman Center for Photomedicine, Massachusetts General Hospital, 65 Landsdowne Street, Cambridge, Massachusetts 02139 USA; Harvard-MIT Division of Health Sciences and Technology, Massachusetts Institute of Technology, Cambridge, Massachusetts 02139 USA

## Abstract

Photonic crystals and band gap materials act as manipulators of light and have a plethora of applications. They are made up of stacks of alternating dielectric constants. This article shows the simulations of an inclined, one dimensional and tuneble photonic crystal, using numerical finite element methods. The photonic crystal was made up of silver nanoparticles embedded in a hydrogel matrix and it has the ability to change and recover its periodicity. A series of factors concerning the geometry of the lattice were tested in order to analyze the efficiency, performance and optimize the properties of the optical sensor. These factors range from the size of the nanoparticles and their density within the stacks, to observing the effect of diffraction angle in readouts.

## Background

Photonic crystals have been widely reported materials with a range of applications such as optical waveguides, [1–3] solar-cells, [4] improved optic fibers, [5] live virus detectors, [6], and medical diagnostics [7]. The research in photonic crystals has advanced significantly over the last decade [8–10]. Photonic crystals can be fabricated from nanoparticle-based structures consisting of alternating stacks of materials possessing different dielectric constants. The structures can be in up to three dimensions; [11] this article will observe the 1D photonic crystals exclusively, wherein the alternating dielectric constant only exists in one direction [12].

The specific configuration used is commonly known as a Bragg mirror and these structures act as efficient reflectors of specific wavelengths of electromagnetic radiation (photonic stopband) [13]. They restrict further propagation of these wavelengths through the lattice and rather they are reflected [14]. The band gap is brought about by constructive interference of reflected waves travelling through the lattice. The photonic band gap effect is highly desirable and can be used in both reflective and antireflective coatings [15], laser cavity end mirrors [16] and functionalized medical devices.

**Fig. 1:**
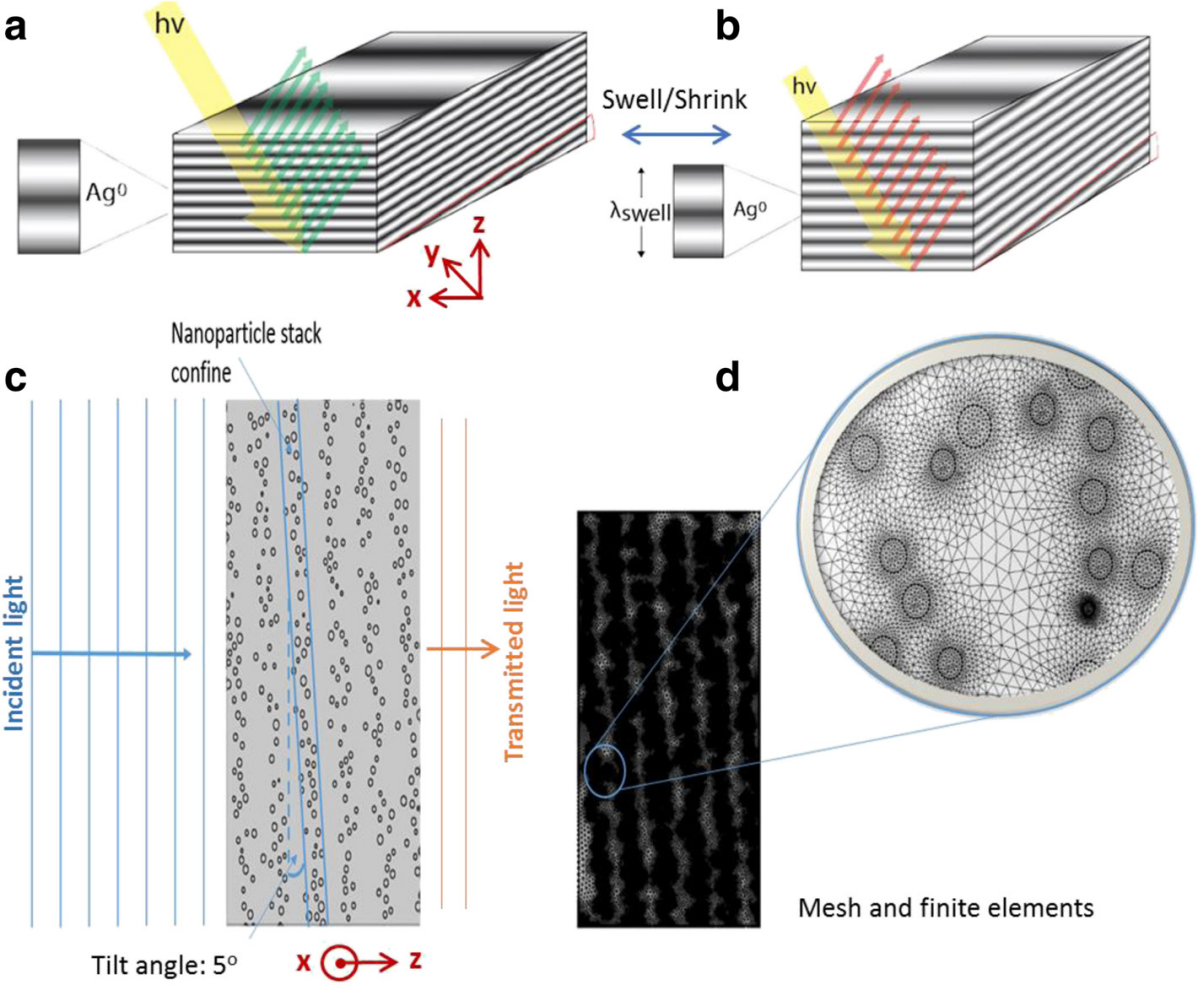
1D photonic crystal sensors. **a**-**b** Schematic of the photonic crystal’s tuning mechanism. **c** Simulation geometry (with 6 stacks) and **d** the associated mesh of finite elements

The photonic crystal that is analyzed consisted of silver nanoparticles which are embedded in poly(hydroxyethyl methacrylate) (pHEMA) hydrogel (Fig. 1a-b) [17]. A relatively cost effective construction method using laser-based photochemical patterning allows creating this tuneable photonic crystal [18, 19]. This crystal changes its periodicity to control specific wavelengths of light, rendering it both tunable and reversible. When compared to other optical filtering techniques such as plasmonic nanoparticle films [20, 21] this device offers dynamic and reversible tunability within the hydrogel volume. Previously we have presented optical biosensors based on 1D photonic crystals [18, 22]. In this work, we analyse a series of simulations run using finite element method to inspect the efficiency and performance, as well as the tunability of Bragg mirror sensors with a 5° slanted lattice akin to blazed transmission gratings.

## Methods

Two-dimensional (2D) modeling of the photonic crystal sensor was achieved using finite element analysis, (COMSOL Multiphysics®. This software was used in conjunction with MATLAB in order to generate the nanoparticle-based structures, which were utilized to simulate different model geometries. A MATLAB code was written to enable the construction of the 1D photonic crystal sensor. The code was set up to generate randomly sized nanospheres distributed within periodic layers. The nanospheres’ positions within the layers were defined such that vertically they were normally distributed but in the horizontal axis, position was given by a normal random distribution where the average position was fixed at a distance equivalent to the lattice constant. The nanospheres therefore were effectively forced to take a position within a rectangular domain (layers), defined by these aforementioned conditions (Fig. 1(c)). The radii of the nanospheres (representative of silver nanoparticles) were also given by a normal random distribution to mimic fabricated devices [17, 23].

The code allowed for the control of the average radius of the nanospheres where the initial run used a value of radius r = 9 nm with a standard deviation of 2 nm. The wavelength was selected to reflect λ = 450 nm at its maxima and refractive index of the hydrogel medium - that the nanoparticles were embedded in – was set to n = 1.512. This allowed for the calculation of the lattice constant where *l* = λ/*2n*. Other controllable parameters included number of nanospheres within each stack and number of stacks.

The generated nanoparticle structures were then imported into COMSOL, rotated by 5° anticlockwise and then enclosed by a square computational domain (Fig. 1). The overall geometry represented a cross-section of the photonic crystal structure (Fig. 1a-b), consisting a stack of layers periodic only in the z direction. The layers extend in the x-y directions; hence, 2D cross-section model is a reasonable simulation geometry of the device. 3D simulation may be more accurate, but it requires much higher time and computational capabilities.

Using the Drude model of permittivity, the refractive index of silver nanoparticles was modeled [24] where both real and imaginary components were provided and assigned to the nanosphere domains. Following the addition of the material properties, scattering boundary conditions were applied around the crystal. Figure 1c shows the entry line for electromagnetic waves moving incident on the crystal from left to right through the stacks of nanospheres. A mesh size defined by COMSOL as “finer” was used throughout; possessing an average element size of roughly 5 nm (Fig. 1d). This structure, defined component was then studied with a parametric sweep, allowing a range of wavelengths (400–900 nm) to be solved in a single run of a simulation, with a 5 nm step size. Boundary integration was performed on the right hand boundary, inspecting “power outflow, time average”, to obtain the plots for a power transmissions against wavelengths. The transmission spectra were normalized by dividing with peak intensity. The simulation process was repeated with the six sets of geometries and the transmission spectra were plotted. Throughout the six simulations, there were three key changes in performance of the photonic crystal although they did not necessarily all exhibit the three changes. These changes consisted of an induced red shift, a wider band gap and also the level of normalized transmission increased in some cases, in turn reducing the peak reflectivity.

All of the simulations were performed with the lattice angled at 5° from the surface plane of the photonic crystal. The comparative effect of having this lattice arrangement is negligible to having the lattice parallel to the long sides. With no noticeable tainting in efficiency or performance between these two configuration, 5° structure offers a reasonably efficient configuration [23]. Without the tilted lattice, the sensor has no practical application since the diffracted light interferes with the incident light.

In the fabricated sensors, the swelling of hydrogel matrix occurs in response to analytes (pH, metal ions, glucose) [25–28]. The swelling not only increases the distance between the nanoparticle layers but also changes the refractive index of the hydrogel. To keep the findings more generic, the index variations have not been considered in this work, rather the focus has been on the optical effects related to the organization of nanoparticles. The refractive index of the hydrogel depends on many factors such as polymer type, molecular weight of the polymer, the concentration of the crosslinker, functional groups, and the concentration-dependent refractive index of the analyte. In this work, we report a generic study by assuming constant index of refraction, temperature and humidity conditions.

## Results and Discussion

### Lattice Spacing Size

**Fig. 2:**
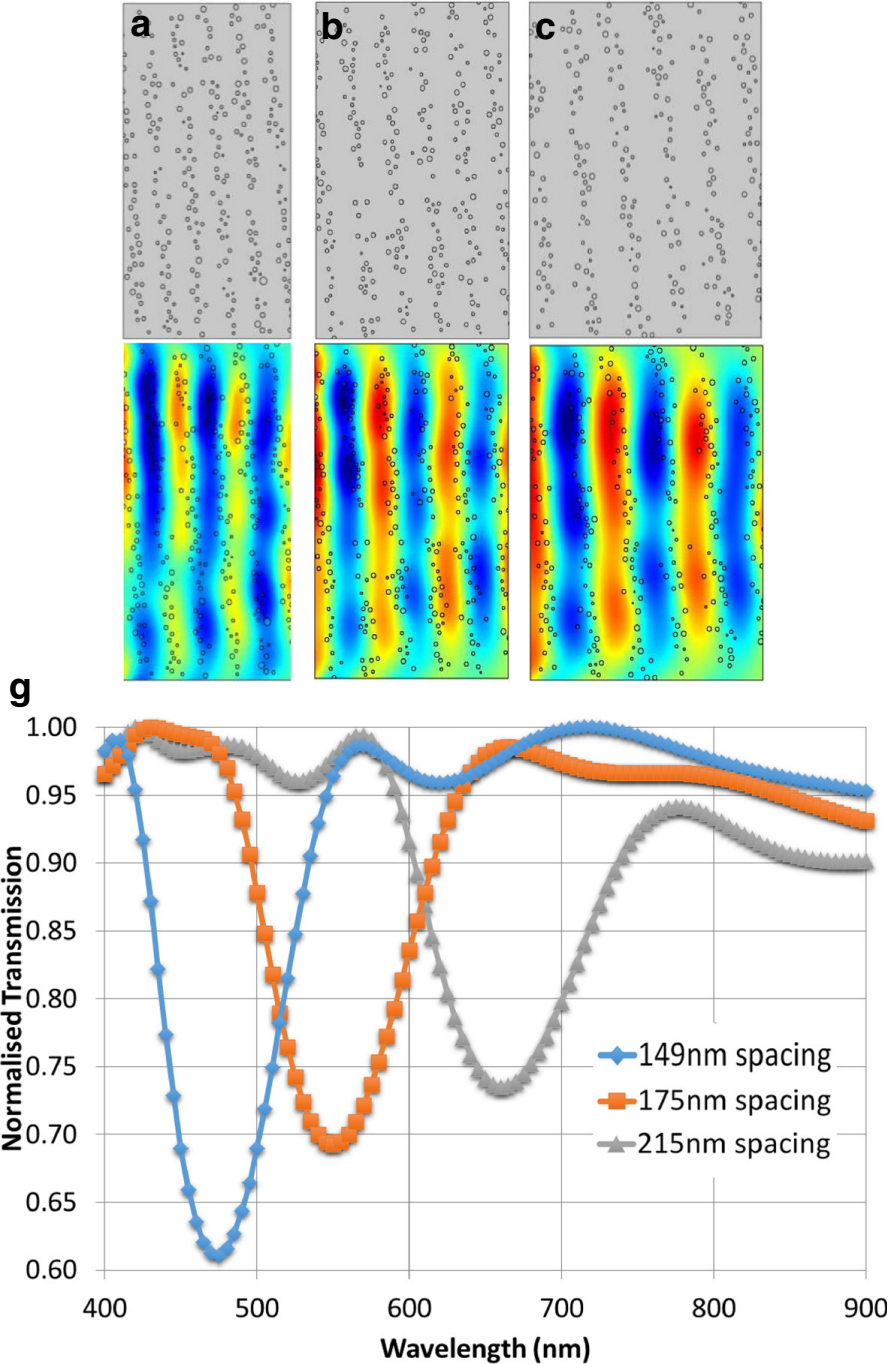
Increasing spacing size l from **a** 149 nm, **b** 175 nm, **c** 215 nm and the associated electric fields of λ= **d** 450 nm, **e** 530 nm, **f** 650 nm propagating electromagnetic waves, respectively. **g** Simulated transmission spectra for increasing lattice spacing

The first parameter inspected was the effective distance between each stack of the lattice. The stack number was set to six, there were 50 particles in each stack and the average particle had 9 nm radius. The wavelengths that should exhibit the most reflection (governing the spacing size) were $\lambda$ =450 nm, 530 nm, and 650 nm. These also equated to corresponding lattice constants ($l$) shown in Fig. 2. The geometries used in the simulation can be seen in Fig. 2(a-c) and coupled with them are their respective electric field plots (d-f). The spacing size between the nanoparticle stacks increases from Fig. 2(a, d) through to Fig. 2(c, f). The associated transmission spectrum is illustrated in Fig. 2(g). Minimum transmission and peak reflectivity of the photonic crystal are located at the minimum point of the dip of each curve where $\lambda \approx 450$ nm for a lattice spacing size of 149 nm. Similarly the minimum point appears at around $\lambda =530$ nm for the middle spacing size of 175 nm and at $\lambda =\sim 650 nm$ for 215 nm spacing. The minimum points should ideally fall at the relevant resonant wavelength, but the above results show a consistent digression (from expected 450 nm, 530 nm, 650 nm) of about 20 nm toward longer wavelengths. The dip represents the band gap and its position translates to the modes of light that are not “allowed” through the crystal. In the case of 149 nm lattice spacing, the modes of light are removed around the 530 nm wavelength to reflect green. As the lattice spacing increases, the band gap shifts toward longer wavelengths.

In order to understand the red shift of the band gap, it must be noted that the whole photonic crystal is expanding as the spacing size increases. The position of the band gap is directly linked to the distance the wave has to travel through the multilayer structure. The band gap appears when returning (reflected) waves destructively interfere with incoming waves – eliminating a specific set of wavelengths (or modes), called a bandgap. If the waves have to travel further, the position at which this band appears will shift upwards. The band gap also experienced a decreased contrast in transmission, as the peak became shallower. The transmission is directly linked to the efficiency of the structure, and so it can also be said that as the lattice expands, the efficiency decreases. This is down to the effective refractive index between the stacks and the medium changing. As the photonic crystal expands, the density of particles within each stack decreases, which reduces the effective refractive index of the stack. As this effective refractive index decreases so too does the depth of the bandgap and the efficiency of the crystal. This is further inspected in the relevant sections.

### Average Nanoparticle Size

**Fig. 3:**
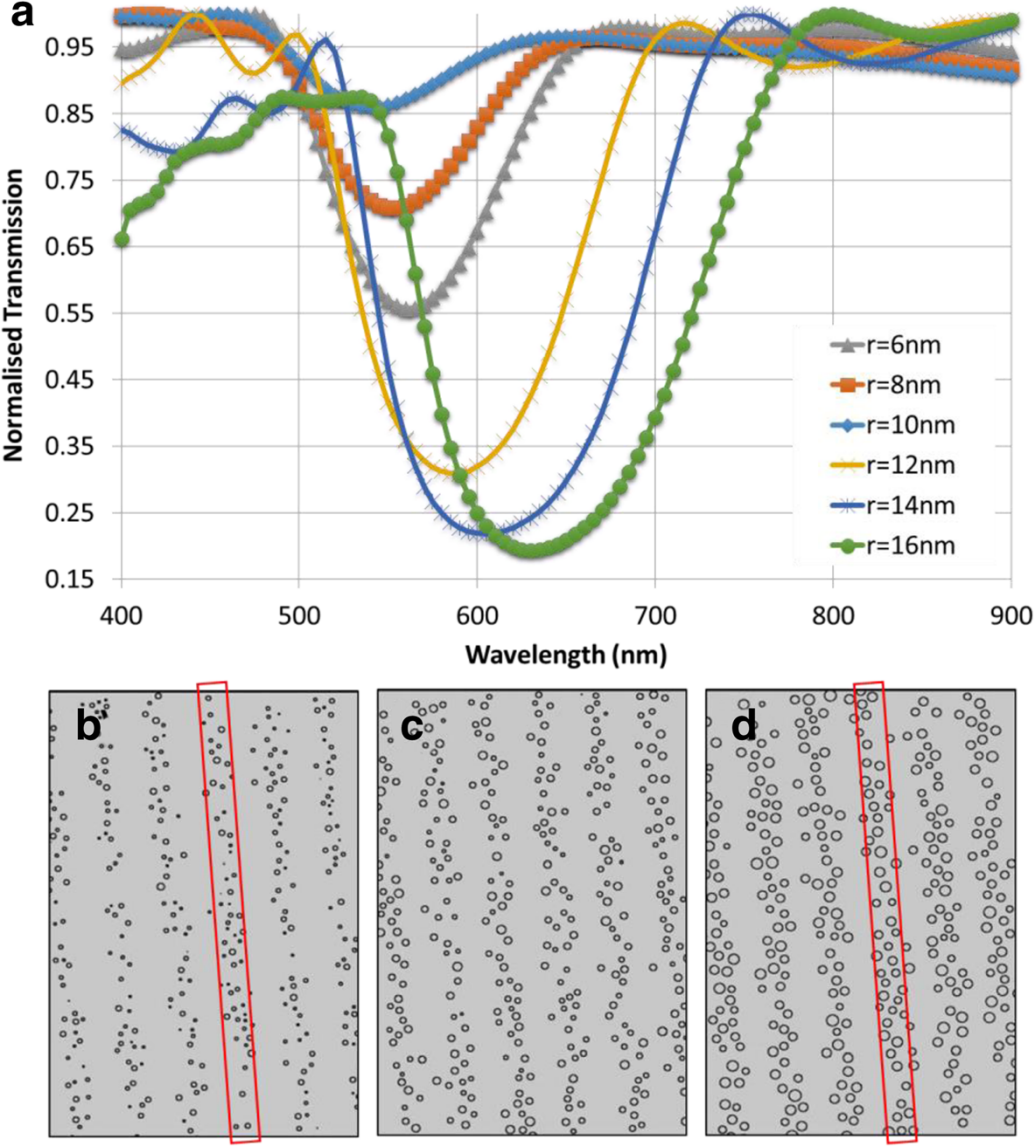
Variation in nanoparticle size. **a** Transmission spectra for increasing average particle size. Geometries with average particle sizes **b** 6 nm, **c** 10 nm, and **d** 14 nm

The next parameter to be inspected was that of the mean size of the nanospheres within the stacks. The spacing size remained constant at $l=175$ nm (suggesting the presence of the band gap at $\lambda =530$ nm) as well as the number of stacks (*i.e.*, 6) with 60 particles per stack. The average sizes to be tested ranged from 6 nm to 16 nm at a 2 nm interval. The six required geometries were constructed with parameters listed in “Lattice Spacing Size” section. Figure 3 shows the transmission spectrum and the depth of the dip (representing the intensity of the reflection band) increasing as the average particles size increases. The increase in average particle size resulted in increased intensity and bandgap width, as well as a red shift. This effect can be explained by the scattering from silver nanoparticles. As the average nanoparticle size increases, the plasmonic resonance peak red shifts and so does the scattering peaks. This phenomenon is observed in the form of the broad and low transmission (dips) [29, 30].

The broadened bandgap may also be due to the operating principle of the MATLAB code - used for creating the structures, in that the centers (not the perimeters) of the particles were confined to the stacks by a rectangle of fixed size. As the particle size increased, the code had to compute positions to fit all the particle’s centers within this same rectangle. This effect was two fold; the effective refractive increased since a larger area of the stack was filled with particles (rather than the hydrogel matrix), and also that the effective spacing size between the stacks decreased, as sections of the particles existed outside of the rectangle (Fig. 3(d)). Unlike the previous case, the lattice remains a fixed size and as the particles get larger, more area within the stack is filled. This causes an effective elevation of dielectric contrast and effective refractive index, increasing the intensity. Fig 3(d) shows the structure where the average particle size was large. There is little uniformity in the width of each stack. This incurs a slightly different spacing size between each stack and consequently introduces a number of band gaps. These bandgaps are of similar coverage and overlap causing one gap of large bandwidth.

### Number of Nanoparticles per Stack

**Fig. 4:**
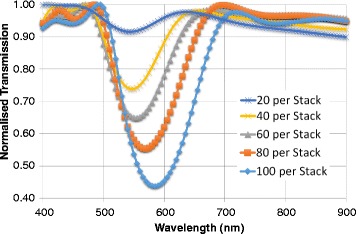
Transmission spectra for increasing the number of particles per stack

The effect of increasing number of particles in each layer was also evaluated. Five geometries were constructed with 6 stacks and an average particle size of 9 nm. A lattice constant of $l=175$ nm was utilized. Initially, there were 20 particles per layer, increasing by 20 each time up to 100 (Fig. 4).

With a growth in the number of particles per layer, the band gap deepens, showing an increased reflectivity. There is, however, a slight red shift present with increasing particles in the stacks. The trough of each transmission curve widens as the stacks are filled with more particles. Adding more nanoparticles to the layers increased the surface coverage of the stack. In covering more of the stack, there is improved uniformity. The effective refractive index contrast sharpens, justifying the stronger reflection band. With a relatively empty configuration of 20 particles per stack, there is a 10 % difference in reflectivity between the stop band and the maximum. In comparison, when the stack is filled up with 100 particles, this value reaches 65 %. The slight red shift can also be accounted for the tighter proximity (or increased density) of nanoparticles increasing plasmonic resonance. The silver nanoparticles have strong absorption and scattering qualities as accounted for by the complex refractive index. The increase in the number of the particles increased the absorptive surface area and scattering, which results in lower light transmission.

### Number of Stacks

The next quality of the photonic crystal to be examined was that of the number of stacks. Most of the parameters were fixed with the values seen previously, whereby the lattice constant was $l=175$ nm, the mean particle size was r = 9 nm and there were 50 particles per stack. The number of layers was varied from 3 to 6.

**Fig. 5:**
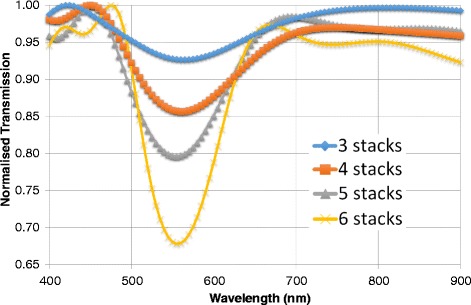
Transmission spectra for increasing number of stacks

Figure 5 shows the different transmission responses to an increasing number of layers within the photonic crystal. As the number of layers (or stacks) increase, a narrower and more intense band gap can be observed. With 6 stacks, there is a 70 % transmission leading to near 30 % reflectivity, whereas 5 stacks shows an 80 % transmission and 20 % reflectivity. There is a consistent band gap positioning – in terms of the position on the visible light spectrum ($\lambda \approx 530$ nm). There is a consistent digression from the expected position of the band gap by 20 nm, in the same direction as in the Figs. 3 and 4. Having more stacks results in stronger reflectivity and a slight narrowing of the bandgap width. While the periodicity stayed constant, the entirety of the structures broadened with added stacks. The incident waves will come into contact with more reflected waves and hence a higher level of destructive interference will be exhibited, improving reflectivity.

### Increasing the Average Size of Particles Across Stacks

**Fig. 6:**
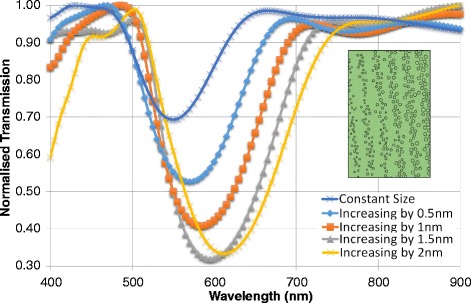
Increasing the average particle size across stacks The inset shows the geometry of increasing average particle size by 2 nm per stack

Variation in particle size represents potential anomalies in the structures. The first of these observed the case of increasing nanoparticle size across the crystal. This is encountered in the fabrication of the crystal whereby an inhomogeneous distribution of particles is obtained due to the uneven reduction of Ag^+^ ions to Ag^0^ nanoparticles using a photographic developer. The inset in Fig. 6 displays a case where the mean particle size ranges from 9 to 19 nm.

To simulate this, standard configuration was applied (Fig. 2), using a lattice constant of $l=175$ nm, but there was an increasing average particle size from one stack to another. The first spectrum was plotted whereby the average particle size started at 9 nm and increased by 0.5 nm from left to right. Figure 6(a) shows a sample geometry of 2 nm increments. The stack on the far left hand side has an average particle size of 9 nm, the second one across is of 11 nm average size, the middle stack 13 nm.

The transmission spectra seen in Fig. 6(b) show many similarities to that of Fig. 5. As the stacks are given larger incremental growths, the band gap positions drop, as in all cases. There is a slight red shift from the constant size to the 1.5 nm increase, but between the last two there is a higher shift. Toward the larger particle size stacks, the spacing between the stacks lessens. The combination of the larger lattice constant and the lack of consistency between spacing sizes accounts for the increased band gap width. The red shift between the 1.5 nm and 2.0 nm particle consisting spectra is likely caused by increased plasmonic resonance/scattering due to the increase in particle size and density. Although the reflectivity is larger when the increment is of 2 nm, the broadness of the band reduces the crystal’s ability to exhibit a selective light reflection.

### Defect Effects

**Fig. 7:**
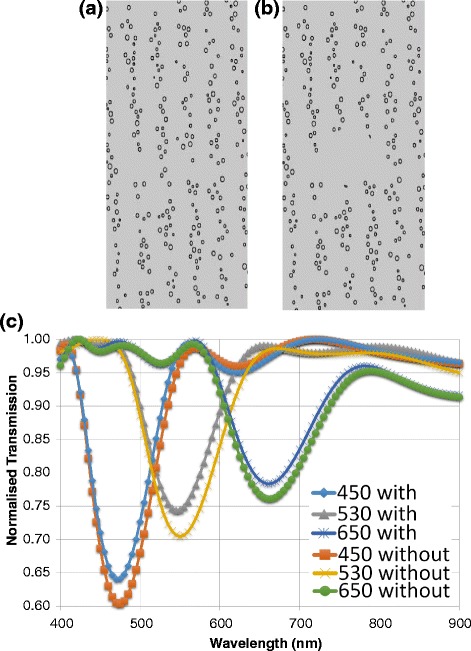
Defect effects in photonic crystals. **a**-**b** Geometry of l=175 nm with and without defect. **c** transmission spectra for increasing spacing size (nm) with and without a defect

Another type of anomaly is the effect of a defect. A defect was introduced into the first simulation (of increasing spacing size) in the form of a empty region in the centre of the lattice, of the same refractive index as the hydrogel medium (Fig. 7a,b). The introduction of a defect into the photonic crystal decreases the normalised transmission in the band gap (Fig. 7c). This could be due to the increased random scattering from the defect. Light is scattered in all directions and less light is transmitted in the normal propagation direction.

### Varying Tilt Angle

**Fig. 8:**
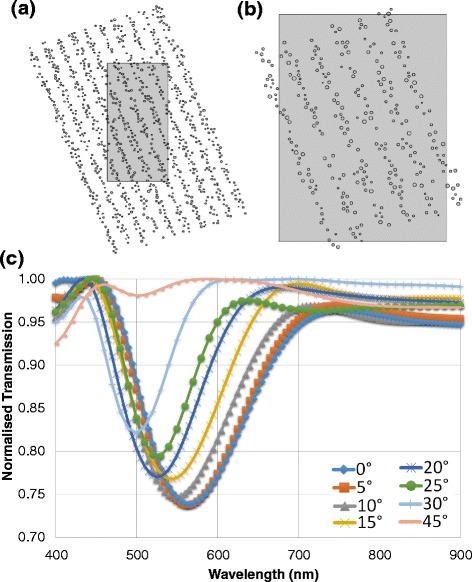
Varying tilt angle of the photonic crystal. **a** Lattice arrangement(particles outside of the medium removed), **b** The consequence of using a regular lattice (large gaps present). **c** Transmission spectra for increasing the lattice tilt angle

Simulations were performed to understand the effect of increasing the lattice tilt angle has on performance and efficiency of the photonic crystal. Thus far, it has only been gauged that 5° shows no real difference in performance (barring the slight error in the dip position). A photonic crystal was generated that could be rotated while maintaining key geometrical properties. The MATLAB code was modified such that it produced a structure with many layers and of excess height but with a standard 9 nm average nanoparticle radius and a lattice constant of $l=175$ nm. A graphic illustration of why the excess height was needed can be seen below in Fig. 8. Empty spaces would be left if the regular structure was used (Fig. 8(b)). By using this technique, a consistency in geometries was obtained. These geometries were tested in the same manner as before and produced a complex set of results.

Figure 8(c) suggests that a more obtuse slant in the lattice should result in a blue shift and should reduce the intensity. Previous findings suggest that rather a red shift should be experienced. As the lattice rotates relative to the centre of the photonic crystal, the effective spacing and distance that the plane wave travels increases. However, the response is the opposite. One possible reason for the blue shift can be that as the tilt angle increases the layers of nanoparticles no longer act as a photonic crystal but rather act as a blazed grating. This grating may reflect the wavelengths according to title angles rather than lattice spacings. The intensity decrease on the other hand, matches up to the theory that as the spacing grows the intensity decreases. It was anticipated that the slant angle would account for the slight discrepancy in the position of the band gap from the expected in Fig. 8c. There was a consistent red shift of 20 nm from the projected position of the band gap. Figure 8(c) shows that no shift in band gap position is experienced between 0° and 5° slants. The discrepancy may be accounted for as a systematic error in the generation of the structure in MATLAB. The code used to determine the lattice spacing (affecting band gap position) may not yield a precise mesh. The consistency of the error suggests that it should be almost neglected and the band gap positions can be assumed to be in the correct position (20 nm towards the blue end of the spectrum).

**Fig. 9:**
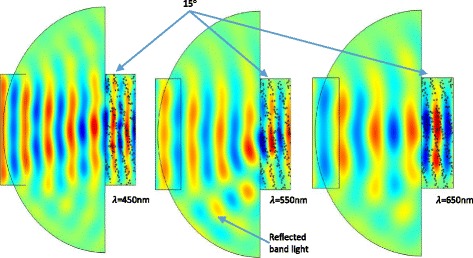
Electric field plots for a 15° slanted photonic crystal with varying wavelengths. The red wave fronts represent maximum power and the dark blue a minimum

An alternative approach to find the effect of varying angle was proposed where an electric field was plotted to show the efficiency of the reflection band light. A region of air was added to the geometry in order that the reflected band gap light could be observed. The geometry was tested with a range of angles (Fig. 9). Light was incident from the far left boundary and all exterior edges were set as scattering boundary conditions. The photonic crystal was of the same configuration as in the previous simulation. At a 15° slant, the photonic crystal resonates at 550 nm. This is evidenced by that fact that the reflected, angled light is most defined at this wavelength; and is also in agreement with the position of the band gap in Fig. 8. The intensity decreases at 450 nm and 650 nm where barely any reflected light is seen. As the wavelength shifts from the resonant frequency, the intensity of the reflected light diminishes.

## Conclusion

We presented simulations to determine the efficiency and performance of a 1D, nanoparticle-based photonic crystal with a 5° slanted lattice angle. The simulations had negligible systematic errors, transposing the position of the band gap by 20 nm in the red direction. This error was also present in the unslanted geometry. The simulations have showed that by altering certain parameters the reflected diffraction can be controlled – including position of band gap (and diffracted colour), efficiency of the reflection and the bandwidth. The position of the band gap and consequent resonant or reflected light was controlled primarily by the spacing size between the nanoparticle stacks. The efficiency can be also increased through adding extra stacks to the multilayer structure. Bandwidth can also be narrowed by increasing the particle density. The simulated models allow for the optimisation of 1D photonic crystals based holographic sensors and other tunable light-filtering applications.
